# Supplementary material for: ETHIAD: A novel explainable model for detecting illicit accounts on Ethereum
Source: PLoS One. 2025 Dec 11;20(12):e0338366. doi: 10.1371/journal.pone.0338366 (PMC12698024; doi:10.1371/journal.pone.0338366)
Supplement: S1 Appendix — (DOCX) [file pone.0338366.s001.docx]

# Appendix A

**Table A1.** Complete list of the 42 extracted features.

| No | Feature name | Feature Explanation | Feature type |
| --- | --- | --- | --- |
| 1 | Avg_min_between_sent_tnx | Average time between sent transactions for account in minutes | Integer |
| 2 | Avg_min_between_received_tnx | Average time between received transactions for account in minutes | Integer |
| 3 | Time_Diff_between_first_and_last(Mins) | Time difference between the first and last transaction | Integer |
| 4 | Sent_tnx | Total number of sent normal transactions | Integer |
| 5 | Received_tnx | Total number of received normal transactions | Integer |
| 6 | Number_of_Created_Contracts | Total Number of created contract transactions | Integer |
| 7 | Unique_Received_From_Addresses | Total Unique addresses from which account received transactions | Integer |
| 8 | Unique_Sent_To_Addresses | Total Unique addresses from which account sent transactions | Integer |
| 9 | Min_Value_Received | Minimum value in Ether ever received | Double |
| 10 | Max_Value_Received | Maximum value in Ether ever received | Double |
| 11 | Avg_Value_Received | Average value in Ether ever received | Double |
| 12 | Min_Val_Sent | Minimum value of Ether ever sent | Double |
| 13 | Max_Val_Sent | Maximum value of Ether ever sent | Double |
| 14 | Avg_Val_Sent | Average value of Ether ever sent | Double |
| 15 | Min_Value_Sent_To_Contract | Minimum value of Ether sent to a contract | Double |
| 16 | Max_Value_Sent_To_Contract | Maximum value of Ether sent to a contract | Double |
| 17 | Avg_Value_Sent_To_Contract | Average value of Ether sent to contracts | Double |
| 18 | Total_Transactions(Including_Tnx_to_Create_Contract) | Total number of transactions | Integer |
| 19 | Total_Ether_Sent | Total Ether sent for account address | Double |
| 20 | Total_Ether_Received | Total Ether received for account address | Double |
| 21 | Total_Ether_Sent_Contracts | Total Ether sent to Contract addresses | Double |
| 22 | Total_Ether_Balance | Total Ether Balance following enacted transactions | Double |
| 23 | Total_ERC20_Tnxs | Total number of ERC20 token transfer transactions | Integer |
| 24 | ERC20_Total_Ether_Received | Total ERC20 token received transactions in Ether | Double |
| 25 | ERC20_Total_Ether_Sent | Total ERC20 token sent transactions in Ether | Double |
| 26 | ERC20_Total_Ether_Sent_Contract | Total ERC20 token transfer to other contracts in Ether | Double |
| 27 | ERC20_Uniq_Sent_Addr | Number of ERC20 token transactions sent to Unique account addresses | Integer |
| 28 | ERC20_Uniq_Rec_Addr | Number of ERC20 token transactions received from Unique addresses | Integer |
| 29 | ERC20_Uniq_Rec_Contract_Addr | Number of ERC20 token transactions received from Unique contract addresses | Integer |
| 30 | ERC20_Avg_Time_Between_Sent_Tnx | Average time between ERC20 token sent transactions in minutes | Integer |
| 31 | ERC20_Avg_Time_Between_Rec_Tnx | Average time between ERC20 token received transactions in minutes | Integer |
| 32 | ERC20_Avg_Time_Between_Contract_Tnx | Average time ERC20 token between sent token transactions | Integer |
| 33 | ERC20_Min_Val_Rec | Minimum value in Ether received from ERC20 token transactions for account | Double |
| 34 | ERC20_Max_Val_Rec | Maximum value in Ether received from ERC20 token transactions for account | Double |
| 35 | ERC20_Avg_Val_Rec | Average value in Ether received from ERC20 token transactions for account | Double |
| 36 | ERC20_Min_Val_Sent | Minimum value in Ether sent from ERC20 token transactions for account | Double |
| 37 | ERC20_Max_Val_Sent | Maximum value in Ether sent from ERC20 token transactions for account | Double |
| 38 | ERC20_Avg_Val_Sent | Average value in Ether sent from ERC20 token transactions for account | Double |
| 39 | ERC20_Uniq_Sent_Token_Name | Number of Unique ERC20 tokens transferred | Integer |
| 40 | ERC20_Uniq_Rec_Token_Name | Number of Unique ERC20 tokens received | Integer |
| 41 | ERC20_Most_Sent_Token_Type | Most sent token for account via ERC20 transaction | String |
| 42 | ERC20_Most_Rec_Token_Type | Most received token for account via ERC20 transaction | String |
