# Supplementary material for: ETHIAD: A novel explainable model for detecting illicit accounts on Ethereum
Source: PLoS One. 2025 Dec 11;20(12):e0338366. doi: 10.1371/journal.pone.0338366 (PMC12698024; doi:10.1371/journal.pone.0338366)
Supplement: Attachment — Submitted filename: Response to Reviewers.pdf [file pone.0338366.s003.pdf]

ETHIAD: A novel explainable model for detecting illicit accounts on Ethereum (Paper ID：PONE-D-24-49589)

Response to Reviewers

We sincerely thank the reviewers and the handling editor for their positive assessment of our work and for the constructive comments provided to improve the quality of the manuscript. We have carefully considered all suggestions and revised the paper accordingly. Our point-by-point responses to the reviewers' and editor's comments are presented below.

**Reviewer #1: [Comment incorporated]**
**Q1:** Your manuscript presents a significant advancement in fraud detection within Ethereum networks. The use of ADASYN and Lasso for preprocessing and the SHAP framework for explainability are particularly commendable. **I recommend discussing challenges encountered during model development and the implications of SHAP values more thoroughly.**<br>**A1:** In response to the comment regarding the **"challenges encountered during model development"** , the authors have made the following revisions:<br>(1) In the third paragraph of Section **"1 Introduction"**, we now provide a clearer and more explicit description of the three major challenges currently faced in illicit account detection. For example:<br>(2) In the fourth paragraph of Section **"1 Introduction"** and the subsequent content, we revised and refined the narrative to ensure that the main contributions of this study directly correspond to the three challenges outlined earlier. This revision further highlights the depth and novelty of our research.<br>(3) In Section **"2 Literature review"**, we improved the summary of prior work to more clearly emphasize the remaining "challenges" in current research and to create a more coherent transition to the distinct contributions of this study.<br>**A2:** In response to the request to **"discuss the implications of SHAP values more thoroughly"**, the authors made the following revisions:

(1) In Section **"3 Model framework and methodology"**, we strengthened the introduction of the SHAP framework and integrated it more closely with the problem of illicit account detection. This helps readers better understand the underlying principles of using SHAP for model interpretability.

(2) In the second paragraph of Section **"5.1 Analysis of the main factors influencing illicit accounts"**, we expanded the explanation of how SHAP summary plots interpret the ETHIAD model's decisions, thereby making the interpretive process clearer and more intuitive for readers.

Reviewer #2: [Comment incorporated]

Q1: The manuscript is technically sound and presents a rigorous methodology for developing the ETHIAD model. **However, it could be improved by explicitly stating the research objectives as questions or hypotheses. This would strengthen the scientific framework of the study and facilitate understanding for readers. The data used appear to align with the objectives, but a more thorough justification of the selected sources would enhance credibility.**

A1: In response to the comment regarding **"stating the research objectives as questions or hypotheses",** the authors have made the following revisions:

(1) The research objectives to be addressed in this study are now clearly stated at the end of both the Introduction and the Literature review sections.

(2) At the beginning of Section **"3.1 Problem definition and ETHIAD framework"**, we explicitly formulate the research problem in the form of a hypothesis.

A2: In response to the comment suggesting that **"a more thorough justification of the selected sources would enhance credibility"**, the authors have made the following improvements:

We clarified the rationale for selecting the dataset by emphasizing that it has been widely adopted as a benchmark in Ethereum fraud-detection research. This justification is now explicitly articulated in several sections of the manuscript, including Section **"2 Literature review"**, Section **"4.2 Data description and feature modeling"**, and Section **"4.3 Experimental comparison with existing work"**.

**Q2:** The use of the XGBoost algorithm combined with SHAP for explainability is well-justified and methodologically sound. **However, a clearer transition between feature modeling and explanatory analysis, as well as clarification of the precise role of each step in the algorithm, would be beneficial for readers less familiar with these techniques.**<br>**A1:** In response to this comment, the authors have elaborated on the role and purpose of each algorithmic component within the ETHIAD framework in Section "**3 Model framework and methodology**" when presenting the model construction pipeline. Furthermore, additional explanations are provided in Section "**4 Experiments and results analysis**" during the description of the experimental procedure, highlighting the critical contribution of each step.
**Q3:** The sources of the variables are mentioned, but a detailed justification of the data selection criteria is missing. Providing an explanation of the relevance and reliability of the data would enhance the transparency and reproducibility of the results.<br>**A1:** In response to this comment, the authors have clarified in Section **"4.2 Data description and feature modeling"** the data used in this study, the reasons for its selection, and its relevance and suitability for the research. In addition, the descriptions and definitions of the dataset variables are provided in **Appendix A1**, and links to the data and experimental code are supplied to ensure the transparency and reproducibility of ETHIAD.
**Q4:** The manuscript is well-structured and written in standard English. **However, a clearer articulation of the contributions as research objectives or questions right from the introduction would improve readability and the overall impact of the text.**<br>**A1:** The authors have highlighted and clarified the research objectives, questions, and contributions of this study in Section **"1 Introduction"**.

**Reviewer #3: [Comment incorporated]**
**Q1:** The provided dataset link (https://github.com/Lujiarong1203/ETHIAD) is currently invalid, which makes it difficult to verify several of the claims made in the manuscript. **It is important to ensure that the dataset is accessible, either through**

**a valid public link or by providing an alternative means of access, to support the transparency and reproducibility of the study.**<br>A1: The authors have provided the links to the dataset and experimental code in the **"Data and Code Availability"** section at the end of the manuscript.
Q2: The research contribution section should be refined to avoid overclaiming. For instance, the statement **"we introduce the SHAP framework"** could be misleading, as it implies that the authors developed the SHAP framework themselves. A more accurate phrasing such as **"we use the SHAP framework"** or **"we apply the SHAP framework in our analysis"** would better reflect the authors' actual contribution and maintain clarity in presentation.<br>A1: The authors have incorporated this suggestion and made the corresponding revisions in the manuscript.
Q3: The statement "the transparent and open nature of the blockchain's transaction information makes it easy to track transactions" requires further clarification. **The authors should elaborate on what specific features of blockchain transparency facilitate transaction tracking and explain how this property is leveraged within their proposed model or analysis.**<br>A1: The authors have supplemented the manuscript to fully clarify that the publicly available Ethereum transaction records provide essential support for constructing illicit account detection models based on fundamental transaction-level features, and that such features are critical for identifying illicit accounts.
Q4: **The manuscript would benefit from a discussion on the computational complexity of the proposed model compared to state-of-the-art (SOTA) approaches**. Providing such a comparison would help readers understand the efficiency of the proposed method. **It is also important to elaborate on how the model reduces the dependence on manual feature design relative to existing solutions,** as this is a key aspect of innovation and practicality.<br>A1: In response to the comment on **"the computational complexity of the proposed model"**, the manuscript has added a fourth paragraph in Section **"4.3 Experimental comparison with existing work"**, to highlight ETHIAD's advantages in algorithmic efficiency.

**A2:** Regarding **"how the model reduces the dependence on manual feature design"**, additional explanations have been provided in the second paragraph of Section "6 Discussion".
**Q5: The reference to Farrugia et al. [20] requires further detail.** The manuscript does not clearly describe the methods used in their fraud detection model—whether they were based on basic transaction data techniques or more advanced approaches. Including this information would provide better context and strengthen the comparative analysis.<br>**A1:** In response to this comment, the authors have provided a detailed description of Farrugia et al.'s work in Sections 2 ("Literature Review") and 4.3 ("Experimental comparison with existing work"). It is emphasized that Farrugia et al.'s study—both in terms of dataset and methodology—serves as a benchmark for comparison, highlighting ETHIAD's advantages in feature engineering, algorithm optimization, and interpretability analysis.
**Q6: Furthermore, it is unclear whether the proposed method was tested on the same dataset as those used by the SOTA solutions.** Clarifying this point would enhance the credibility of the performance comparison and help readers evaluate the relative effectiveness of the proposed approach.<br>**A1:** This issue is particularly important and had not been explicitly addressed in the original manuscript. Therefore, the authors clarified in Section 4.3 ("Experimental comparison with existing work") that ETHIAD was compared against nine existing models, among which the models in references [20 – 22] were constructed using the same dataset as ETHIAD.
**Q7:** The authors claim that existing datasets suffer from category imbalance, which distorts model performance. **However, no supporting evidence or references are provided to substantiate this statement.** Including empirical data or literature support would significantly strengthen this argument.<br>**A1:** In response to this comment, the authors clarified in Section 4.2 ("Data description and feature modeling"), subsection "(3) Sample Sampling", the impact of class imbalance in the dataset on model training, supported by a reference to [35].
**Q8:** Also, the selection of algorithms—Random Forest, LightGBM, and XGBoost—

requires further justification. **The authors should explain why other commonly used algorithms such as Decision Trees, ETH-PSD, AdaBoost, or KNN were not considered, particularly since they have been utilized in related studies.** Including a brief comparison or rationale would help demonstrate the robustness and relevance of the chosen methods.

A1: Regarding the rationale for selecting XGBoost as the core algorithm for ETHIAD, the authors made the following revisions:

(1) In Section 3.2 ("XGBoost algorithm"), the description of XGBoost was updated to highlight its characteristics and advantages in handling high-dimensional, multi-type features typical of Ethereum transaction data.

(2) In Section 4.4 ("Experimental comparison with mainstream models"), a comparative analysis with ten mainstream machine learning models was provided, further demonstrating the reasonableness of choosing XGBoost.

Q9: **Additionally, several figures referenced in the text appear to be missing from the manuscript.** The authors should ensure that all figures are included, properly labeled, and clearly referenced within the text to support the narrative.

A1: As the authors have formatted and edited the manuscript in accordance with the submission guidelines, all figures have been uploaded separately as individual files to the submission system.

Editor: [Comment incorporated]

Dear Editor,

Thank you for providing the opportunity to further improve our manuscript. We have carefully addressed all the requested revisions. The updates are summarized as follows:

(1) The manuscript formatting and layout have been further refined in accordance with the journal's submission guidelines.

(2) The code used in this study has been shared.

(3) Corrections have been made to the Funding Information and Financial Disclosure sections.

(4) The Acknowledgements section has been standardized and revised.
